# A case report of dengue haemorrhagic fever complicated with psoas haematoma requiring blood transfusion

**DOI:** 10.1186/s12879-019-4023-2

**Published:** 2019-05-06

**Authors:** Anne Thushara Matthias, Sanduni Apsara, Apsara Epa

**Affiliations:** 1 Dengue High Dependency Unit, Colombo South Teaching Hospital, Kalubowila, Sri Lanka; 2 Department of Radiology, Colombo South Teaching Hospital, Kalubowila, Sri Lanka

**Keywords:** Dengue fever, Dengue hemorrhagic fever, Muscle haematoma

## Abstract

**Background:**

Dengue fever is a common infection with increasing numbers of patients affected. Muscle haematomas are a rare complication of dengue fever. In most cases haematomas resolve spontaneously.

**Case presentation:**

We report a case of spontaneous psoas muscle haematoma, formed during the critical phase of dengue haemorrhagic fever. A 28-year-old gentleman presented with features of severe dengue and was admitted to the Dengue High Dependency Unit. He was treated with intravenous fluid therapy and supportive measures, and gradually improved initially. However, as the critical phase ended, he suddenly developed pain in the left groin and inguinal region and physical examination was normal. Ultrasound scan revealed a left psoas haematoma. As the patient deteriorated haemodynamically blood was transfused. He recovered without further complication and was discharged home.

**Conclusions:**

Dengue fever is a common tropical infection. Recognizing serious complications such as psoas haematoma presenting as simple complaints such as back pain and inguinal pain are important to prevent mortality.

## Background

Dengue fever is one of the commonest infections affecting a large population of people in Sri Lanka. During the 5 years from 2012 to 2016, nearly 800 Dengue cases were reported per week [1]. The Dengue fever outbreak in 2017 in Sri Lanka was caused by DENV-2 virus. It is assumed that the 2018 cases were also caused by the same [2]. Dengue has a wide spectrum of symptoms. The severe form of Dengue been dengue shock syndrome is the most dreaded complication of dengue. During the course of dengue fever all organs in the body can be affected resulting in numerous complications and this is termed Expanded Dengue Syndrome (EDS) [3]. The EDS is increasingly reported. Musculoskeletal system is also involved in Dengue fever. Though dengue fever is known to cause musculoskeletal problems such as arthralgia and myalgia. The bleeding complications of dengue are mostly mucosal such as epistaxis and petechiae. Severe dengue can cause bleeding complications such as haematemesis and malaena [4, 5]. Spontaneous large muscle haematoma formation in Dengue Hemorrhagic fever (DHF) is rare.

## Case presentation

A previously healthy 38-year-old, from Colombo, presented to the Dengue HDU with generalized body ache and high fever (highest recorded temperature 39·4 °C) for 3 days. On physical examination, he was found conscious (Glasgow Coma Scale 15), dehydrated with a heart rate 100/min, blood pressure 100/60 mmHg. There was no rash or active bleeding. Other general and systemic examinations revealed no abnormality. The working diagnosis on admission was dengue fever. On day 3, Dengue haemorrhagic fever (DHF) was diagnosed based on a right sided pleural effusion, gall bladder wall oedema and free fluid in hepatorenal pouch and the patient was started on management as per the critical phase. Critical phase was started as the patient had evidence of leaking with a right sided pleural effusion. Oral fluid therapy as per WHO guideline were commenced.

On completion of the critical phases he complained of a severe left sided groin and inguinal region pain. On examination the skin looked normal and there was no tenderness but he had severe pain when flexing the left thigh. There was no visible swelling. All the pulses of the lower limb were present and neurological examination was normal. The timeline of events is given in Table 1.

**Table 1:** Clinical events with timeline

Day of illness	Platelet count	Haemoglobin	Haematocrit	Clinical event
Day 3	15,000	13.9	42.5	Critical phase started
Day 5	13,000	14.0	43.0	Critical phase ended
Day 6	12,000	12.6	46	Complained of left hip pain
Day 7	12,000	12.3	46	USS: Haematoma on left psoas
Day 8	26,000	11.8	34	Blood transfusion. USS; No change in size of haematoma Meropenam started after blood culture
Day 9	73,000	12.5	46	CRP 16. Fever settled.
Day 11	25,000	12.8	47	Discharged

Significant initial laboratory investigations showed haemoglobin (Hb) 13.2 g/L (13-15 g/L), haematocrit (Hct) 44%, total leukocyte count (TLC) 4·94 K/μL, platelet count (PC) 15 K/μL, alanine aminotransferase (ALT) 88.4 U/L(Normal < 40), aspertate aminotransferase (AST) 95 U/L (Normal < 40), serum sodium 130 mmol/L(135–145), and C-reactive protein (CRP) 37·7 nmol/L (< 6). PT 14 (11 to 13.5 s), APTT 36 s (30–40). Blood picture: evidence of viral infection. Clinical suspicion of DHF was confirmed by positive dengue-NS1-antigen and anti-dengue-antibodies (IgM and IgG).

**Fig. 1:**
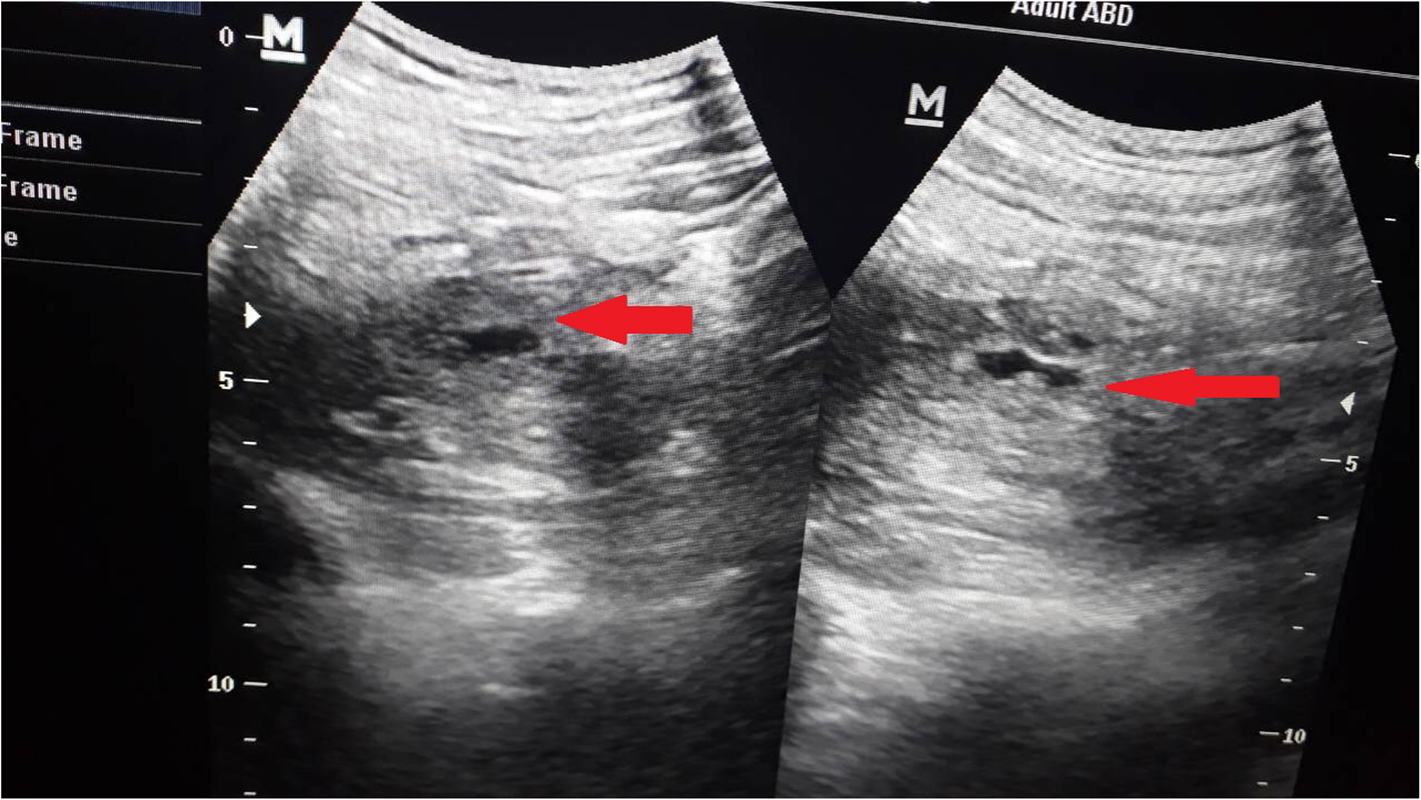
Haematomas seen in the left psoas muscle

**Fig. 2:**
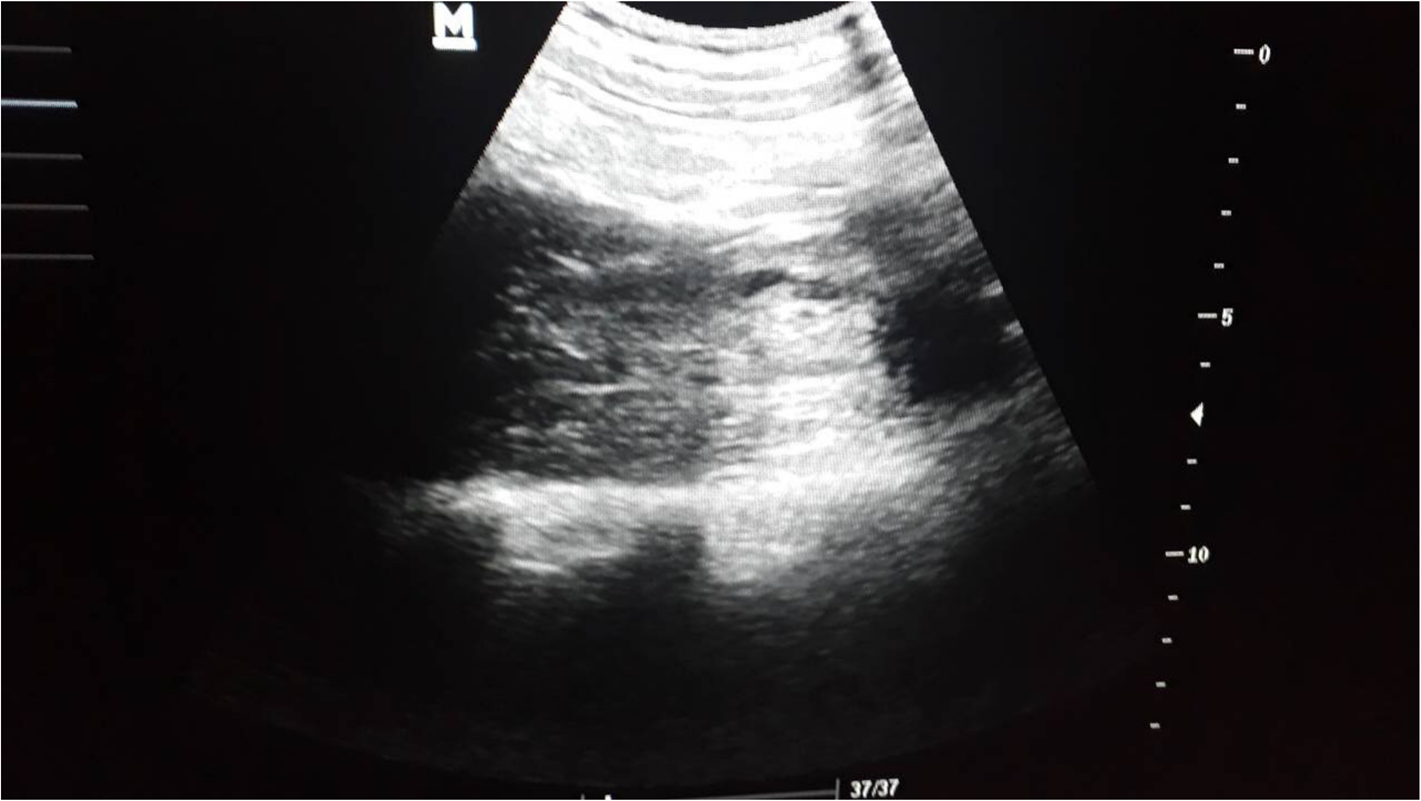
Haematomas seen in the left psoas muscle

After the sudden development of the left thigh pain and an urgent USG showed a hypoechoic area involving the superficial fibers of the middle part of the left psoas. Vascularity is not increased. Rest of the psoas appear normal. Hip joint is normal. Appearances are due to bleeding in to the left psoas with possible secondary infection. No fluid in the compartment to aspirate (Figs. 1 and 2). Platelet on the day of development of haematoma was 12 K/μL, which was the lowest, recorded during his admission. The PT time 14 (11 to 13.5 s), APTT 36 s (30–40), fibrinogen was 2 (1.5-4 g/L). The patient was continued on critical monitoring. As the pain continued the patient developed tachycardia and a drop in PCV was noticed. He also developed a fever spike with CRP rising to 95. A second US: Showed an ill-defined hypoechoeic area seen in the left psoas region without increased vascularity compatible with previously identified Left psoas haematoma without expansion or worsening. The patient was transfused with 5 ml/kg of red cells due to reduction of PCV and tachycardia. The tachycardia settled and hematocrit picked up. He was also given Meropenum as there was evidence of secondary infection of the haematoma as the CRP rose to 75 and there was a secondary spike of fever. No further treatment or support was required. The patient improved gradually on the following days, and was discharged home on the eleventh day of admission, with the diagnosis of severe dengue with atypical manifestation (spontaneous left psoas haematoma). He was followed-up after 1 month and was well and the repeat US scan showed no haematoma.

## Discussion and conclusions

The clinical features of Dengue fever and DHF are variable. DHF is becoming more common in the world over and physicians must be careful of its rare manifestations like muscle haematomas. These muscles haematomas can be in varied muscles: iliopsoas, psoas, retroperitoneal. To our knowledge only one previous case of psoas haemtoma has been reported [6]. Two cases of ilio-psoas haematomas [7–9] and one case of calf haematomas [10] have been reported. These consequences of haematoms can be varied. They could be asymptomatic, could lead to shock or may compress nerves leading to compression syndrome and retroperitoneal haematomas can be life threatening.

The exact pathogenesis of bleeding in dengue fever is still unknown. The postulated mechanisms of bleeding is thought to be autoimmunity against the human cells due to viral infection [8, 11]. This autoimmune response causes endothelial dysfunction, increased vascular permeability and thrombocytopenia. The exact pathogenesis of bleeding in dengue is not completely understood. Studies done in the past have demonstrated, low circulating levels of proteins C, S, and antithrombin III due to leakage of these proteins through the vascular endothelium [12] .In the fibrinolytic system, slightly increased levels of tissue-plasminogen activator (t-PA), plasminogen activator inhibitor (PAI-1) and decreased thrombin-activatable fibrinolysis inhibitor (TAFI) have been reported [13]. Thromboelastometry in patients with DHF have shown decrease of clotting factors and low platelet counts. The activation of the fibrinolytic mechanism may be a cause for spontaneous muscle haematomas in Dengue Haemorrhagic fever. There is some evidence of development of antibodies which might be cross reactive to plasminogen, that could be worsening the haemorrhage in DHF [14]. We postulate that similar mechanisms would have led to the occurrence of haematoma in our patient as all coagulation tests were normal apart from platelet count.

The importance of recognizing muscle haemtomas is the prompt start of vigilant monitoring to prevent the patient form going into shock, by timely intervention of fluid resuscitation, blood transfusion if indicated and evacuation rarely if causing compression syndromes. Our patient did not have any neurological complications.

Most muscle haematomas in Dengue Haemorrhagic fever can be managed conservatively as most cases resolve spontaneously. Few cases have been managed with platelet transfusion as platelets have been very low and as in our case blood transfusion as the patient was continuing to bleed in to the muscle resulting in PCV drop and hemodynamic instability.

## Abbreviations

ALT: Alanine aminotransferase
AST: Aspertate aminotransferase
CRP: C-reactive protein
DHF: Dengue Haemorrhagic Fever
EDS: Expanded Dengue Syndrome
Hb: Haemoglobin
Hct: Haematocrit
HDU: High Dependency Unit
PC: Platelet count
PCV: Packed Cell Volume
TLC: Total leukocyte count
